# Peroxiredoxin 3 has a crucial role in the macrophage polarization by regulating mitochondrial homeostasis

**DOI:** 10.1186/s12931-024-02739-9

**Published:** 2024-03-02

**Authors:** Wenhui Huang, Lianfang Wang, Zhipeng Huang, Zhichao Sun, Bojun Zheng

**Affiliations:** 1 Department of Critical Care Medicine, The Second Affiliated Hospital of Guangzhou University of Chinese Medicine, Guangzhou, China; 2 Department of Respiratory and Critical Care Medicine, Guangdong Provincial Key Laboratory of Major Obstetric Diseases, Guangdong Provincial Clinical Research Center for Obstetrics and Gynecology, The Third Affiliated Hospital of Guangzhou Medical University, Guangzhou, China; 3 Department of Respiratory and Critical Care Medicine, Guangxi Hospital Division of The First Affiliated Hospital, Sun Yat-sen University, Guangxi, China; 4 Dongguan Hospital of Integrated Chinese and Western Medicine, Dongguan, China; 5 The Second Affiliated Hospital of Guangzhou University of Chinese Medicine, Guangzhou, China

**Keywords:** Macrophage polarization, Sepsis-associated acute lung injury, PRDX3, Metabolic reprogram, Mitochondrial function

## Abstract

Acute lung injury (ALI) is one of the life-threatening complications of sepsis, and macrophage polarization plays a crucial role in the sepsis-associated ALI. However, the regulatory mechanisms of macrophage polarization in ALI and in the development of inflammation are largely unknown. In this study, we demonstrated that macrophage polarization occurs in sepsis-associated ALI and is accompanied by mitochondrial dysfunction and inflammation, and a decrease of PRDX3 promotes the initiation of macrophage polarization and mitochondrial dysfunction. Mechanistically, PRDX3 overexpression promotes M1 macrophages to differentiate into M2 macrophages, and enhances mitochondrial functional recovery after injury by reducing the level of glycolysis and increasing TCA cycle activity. In conclusion, we identified PRDX3 as a critical hub integrating oxidative stress, inflammation, and metabolic reprogramming in macrophage polarization. The findings illustrate an adaptive mechanism underlying the link between macrophage polarization and sepsis-associated ALI.

**Supplementary Information:**

The online version contains supplementary material available at 10.1186/s12931-024-02739-9.

## Introduction

Acute lung injury (ALI) increases the morbidity and mortality associated with sepsis [1–3]. The rapid recruitment and activation of proinflammatory macrophages in the lungs is a pivotal process in the pathogenesis of sepsis-induced ALI [4–7]. The unfavorable prognosis of ALI is attributed to limited treatment options, thereby creating an urgent and unmet medical need.

Macrophages are a crucial component of innate immunity and have high plasticity [8, 9], which can present as divergent phenotypes and functions. Macrophages can develop into classically activated macrophages (M1 type), and alternatively activated macrophages (M2 type) in response to different environmental stimuli [10]. Activation of M1 macrophages with various inflammatory stimuli causes substantial metabolic alternations, such as causing a changed from oxidative phosphorylation (OXPHOS) to glycolysis in an inflammatory environment [11]. M2 macrophages obtain energy from fatty acid oxidation and OXPHOS [12, 13]. Many transcription factors, such as interferon regulatory factors [14] and nuclear transcription factor (NF)-κB [15], play an important role in macrophages polarization. Cellular ROS production mainly depends on the mitochondrial respiratory chain [16]. In the normal conditions, ATP is produced upon the entry of electrons and OXPHOS in mitochondria. M1 macrophages possess a bactericidal function as they produce a large amount of ROS upon the contact with pathogen. At the same time, defected ROS production favours the M2 macrophage polarization and the anti-inflammatory response. Therefore, ROS plays a crucial role in maintaining the M1/M2 macrophages polarization. Studies have shown that in the ALI model, resident macrophages polarize into inflammatory M1 macrophages, and peripheral blood monocytes are subsequently recruited to alveoli and polarize to M1 type in the early stage of ALI [17]. In addition, the number of M1 macrophages in the lungs is much larger than M2 macrophages, and the pro-inflammatory factors such as ROS play a key role in the excessive inflammatory response of ALI. Because of the polarization of macrophages in the development of sepsis-induced lung injury, manipulation of macrophage polarization may be a potential therapeutic strategy.

Peroxiredoxins (PRDXs) are ubiquitous and conserved antioxidant enzymes [18], whose main function is to scavenge reactive oxygen species (ROS). PRDX3 is a mitochondrial antioxidant protein with a conserved catalytic cysteine residue [19], and is the principal peroxidase responsible for approximately 90% of the metabolism of mitochondrial hydrogen peroxide (H_2_O_2_) [18, 20]. Previous studies have reported that PRDX3 can significantly inhibit oxidative stress and attenuate cellular damage [18–20]. Study has also shown that PRDX3-knockout mice accumulate relatively high intracellular ROS levels [21, 22], which increases the severity of lipopolysaccharide (LPS)-stimulated lung injury. In contrast, PRDX3 overexpression in mice protects against injury by preserving mitochondrial function and preventing mitochondrial oxidative damage [23, 24]. However, the potential role of PRDX3 in sepsis has not been clarified, and the mechanism of PRDX3 in macrophage polarization requires study.

In this study, we hypothesized that macrophage polarization participates in sepsis-induced lung injury and examined the mechanism and function of PRDX3 in this pathological process. Our findings indicated that targeting metabolic reprogramming through PRDX3 to alleviate ROS accumulation and induce M2 macrophage polarization might be an effective treatment of sepsis-associated ALI.

## Materials and methods

### Animal models of sepsis

C57BL/6 mice (6–8 weeks old, 20-25 g) were purchased from the Central Animal Care Facility of Guangdong Province Hospital, and the experiments were approved by the Committee on the Ethics of Animal Experiments (Permission No. 2023017). Mice were housed in groups of 6 animals per cage in a specific pathogen-free room with a 12-h light/dark cycle, controlled temperature and humidity, and had free access to water and food.

*Endotoxemia model*. A male wild-type mouse endotoxemia models was induced by intraperitoneal injection of Lipopolysaccharides (LPS; E coli 0111: B4) (Sigma-Aldrich, St. Louis, MO, USA) (10 mg/kg). Twelve hours after LPS injection the mice were euthanized by an overdose of pentobarbital (100 mg/kg). Tissue samples were collected and stored at -80℃ for subsequent analysis.

*Cecal ligation and puncture (CLP) model*. A male wild-type mouse CLP models was induced by cecal ligation and puncture via using a surgical procedure. Anesthesia was induced with sevoflurane (2–4%, inhalation). The cecum was exteriorized from a small midline abdominal incision and ligated with 4 − 0 silk. Then, a 25-gauge syringe needle was used to puncture the cecum. The peritoneum and skin were sutured in turn, and the mice were injected subcutaneously with 1 ml of saline solution. After 48 h, the mice were euthanized by an overdose of pentobarbital (100 mg/kg). Tissue samples were collected and stored at -80℃ for subsequent analysis.

### Histological immunohistochemistry (IHC) analysis

The lung tissues were fixed in 4% paraformaldehyde for more than 24 h. After dehydration and paraffin embedding, the tissues were sliced into 4 μm sections, which were strained with hematoxylin & eosin (H&E) staining and IHC stains, respectively. For IHC staining, the lung section slides were incubated with primary antibodies (dilution at 1:200) against CD206 (18704-1-AP, Proteintech), inducible nitric oxide synthase (iNOS) (22226-1-AP, Proteintech), SOD1 (10269-1-AP, Proteintech), and myeloperoxidase (MPO) (22225-1-AP, Proteintech) according to the manufacturer’s instructions. After staining, the sections were observed with light microscopy.

### Immunofluorescence staining

Lung sections were treated with 4% paraformaldehyde for 15 min and 0.2% triton for 10 min, and were then blocked with 5% goat serum for 60 min at room temperature. Tissue sections were then incubated with primary antibodies (dilution at 1:200) at 4℃ for overnight, and stained with fluorescein isothiocyanate (FITC) (A0562, Beyotime) and coealite594-conjugated secondary antibody (SA00013-4, Proteintech) for 1 h. The sections were then stained with DAPI (4′,6-diamidino-2-phenylindole) (F6057, sigma). The antibodies used were as follows: CD206 (18704-1-AP, Proteintech), Arg-1 (16001-1-AP, Proteintech), iNOS (22226-1-AP, Proteintech), CD68 (28058-1-AP, Proteintech) and PRDX3 (55087-1-AP, Proteintech).

### Cell culture and treatment

Raw 264.7 cells were cultured in Dulbecco’s Modified Eagle Medium (DMEM; GIBCO BRL, Life Technologies, Inc., Rockville, MD, USA) with 10% fetal bovine serum (FBS; GIBCO BRL, Life Technologies, Inc., Rockville, MD, USA) and penicillin/streptomycin (100 U/ml and 100 U/ml, respectively; Sigma-Aldrich) at 37 °C in a 5% CO2 incubator. Cells were treated with LPS (1 µg/ml), IFN-γ (50 ng/ml), H_2_O_2_ (10 µmol/L), Oligo (10 µg/mL), NAC (1 mmol/L), visomitin (20 nmol/L), 3PO (10 µmol/L) and mitoquinone (50 nm). Bone marrow-derived cell from C57BL/6 mice were cultured in DMEM media supplemented with 10% FBS and 1% penicillin/streptomycin and differentiated to bone marrow-derived macrophages (BMDMs) by recombinant murine granulocyte- macrophage colony stimulating factor (GM-CSF) (25 ng/mL; Miltenyi Biotech) for 7 days.

### Western blotting

The cells and lung tissues were washed with 1×PBS, and lysed in RIPA lysis buffer containing protease inhibitors. The cell lysates were separated by SDS-PAGE, transferred to PVDF membranes, and immunoblotted with the indicated antibodies. The antibodies used were (dilution at 1:1000): CD206 (18704-1-AP, Proteintech), Arg-1 (16001-1-AP, Proteintech), iNOS (22226-1-AP, Proteintech), tumor necrosis factor (TNF)-α (60291-1-AP, Proteintech), GAPDH (10494-1-AP, Proteintech), TOM20 (11802-1-AP, Proteintech), PRDX3 (55087-1-AP, Proteintech), COXIV (11242-1-AP, Proteintech), NDUFB8 (14794-1-AP, Proteintech), SDHA (14865-1-AP, Proteintech), UQCR1 (14742-1-AP, Proteintech), and ATP5A (14676-1-AP, Proteintech). Protein bands were visualized by the Odyssey System from LI-COR Biosciences.

### RNA isolation and quantitative real-time PCR

Total RNA was extracted from samples using Trizol reagent, and 1 µg of RNA was reverse transcribed to generate cDNA. The cDNA was subjected to qRT-PCR by using the SYBR Green Master Mix. The relative fold change was calculated by the comparative CT method. The primer sequences are listed in Table 1.

**Table 1:** Sequences of the primers used for this study

Gene	Forward sequence 5′-3′	Reverse sequence 5′-3′
CD206	CCACGGATGACCTGTGCTCGAG	ACACCAGAGCCATCCGTCCGA
Arg-1	GAATGGAAGAGTCAGTGTGG	AATGACACATAGGTCAGGGT
iNOS	AGTCTCAGACATGGCTTGCCCCT	GCTGCGGGGAGCCATTTTGGT
TNF-α	GACCCTCACACTCAGATCATCT	CCTCCACTTGGTGGTTTGCT
PRDX3	TGCTGGCATTGCACTCAGA	ACTTCTCCATGGGTCTCCACAA
GAPDH	AGAGTGTTTCCTCGTCCCGT	ACTGTGCCGTTGAATTTGCC

### Adenovirus vector and cell transfection

The recombinant adenovirus was produced by GenChem Company (Shanghai, China). The amplified PRDX3 fragment was inserted into MCS-3FLAG-EF1a-ZsGreen1-T2A-puromycin, which contains the mouse cytomegalovirus (CMV) promoter. Cells were transfected with lentiviral particles containing enhanced green fluorescent protein (pGC-FU-GEP, lenti-GFP/lenti-NC) or murine PRDX3 (pGC-FU-GEP, lenti-GFP/lenti-PRDX3), according to the manufacturer’s protocol.

### Measurement of intracellular ATP production

Intracellular ATP concentration was detected by an ATP assay kit from Beyotime (S0027), according to the manufacturer’s instructions.

### MitoSOX Red, Mito-tracker Red CMXRos, Mito-tracker green staining

Living cells were incubated with MitoSOX™ Red (2.5 µM, M36008, Invitrogen), Mito-Tracker Red CMXRos (1 µM, C1049B, Beyotime), or Mito-tracker Green (2 µM, C1048, Beyotime) for 15 min at 37℃ in dark. Then the cells were washed with Hank’s Balanced Salt Solution (HBSS)/Ca/Mg and analyzed by confocal microscopy (LSM880, Carl Zeiss).

### Mitochondrial membrane potential assay

Mitochondrial membrane potential changes were detected in RAW 264.7 cells using a JC-1 mitochondrial membrane potential assay kit from Beyotime (C2006). Briefly, JC-1 gathered into J-aggregates in the mitochondrial matrix and showed red fluorescence under conditions of a higher mitochondrial membrane potential, whereas the JC-1 monomer showed a green fluorescence when the mitochondrial membrane potential was lower. After the different treatments, the cells were loaded with JC-1 staining at 37℃ for 20 min. Then the cells were washed with HBSS/Ca/Mg and analyzed by confocal microscopy (LSM880, Carl Zeiss).

### Measurement of the intracellular reduced glutathione/oxidized glutathione (GSH/GSSG), superoxide dismutase (SOD), and malondialdehyde (MDA) production

GSH/GSSG production was detected by a GSH/GSSG assay kit from Beyotime (S0053), the SOD production was detected by a CuZn/Mn-SOD assay kit from Beyotime (S0103), and MDA production was detected by an MDA assay kit from Beyotime (S0131S). All assays were performed according to the manufacture’s protocols.

### Targeted metabolomics analysis

Targeted metabolomics analysis of the metabolites was performed with assistance from the Wekemo Tech Group Co., Ltd. (Shenzhen, China). Briefly, the metabolites were extracted from RAW 264.7 cell macrophages transducted with adv-NC or adv-PRDX3. Analyses were performed using an Ultra High Performance Liquid Chromatography (UHPLC) (Agilent 1290 Infinity LC) coupled to a QTRAP (SCIEX 5500).

### Enzyme-linked immunosorbent assay (ELISA)

Cell culture supernatants were analyzed for IL-1β, TNF-α, and IL-6 using ELISA kits (ThermoFisher, USA), according to the manufacturer’s instructions.

### Statistical analysis

Data were presented as mean ± standard deviation (SD). Intergroup comparisons of mean values were performed using one-way analysis of variance (ANOVA). Data analyses were performed using SPSS version 22.0 software (SPSS Inc., Chicago, IL, USA). Statistical significance was defined as *P* < 0.05.

## Results

### Macrophage polarization occurs in in vivo sepsis models

**Fig. 1:**
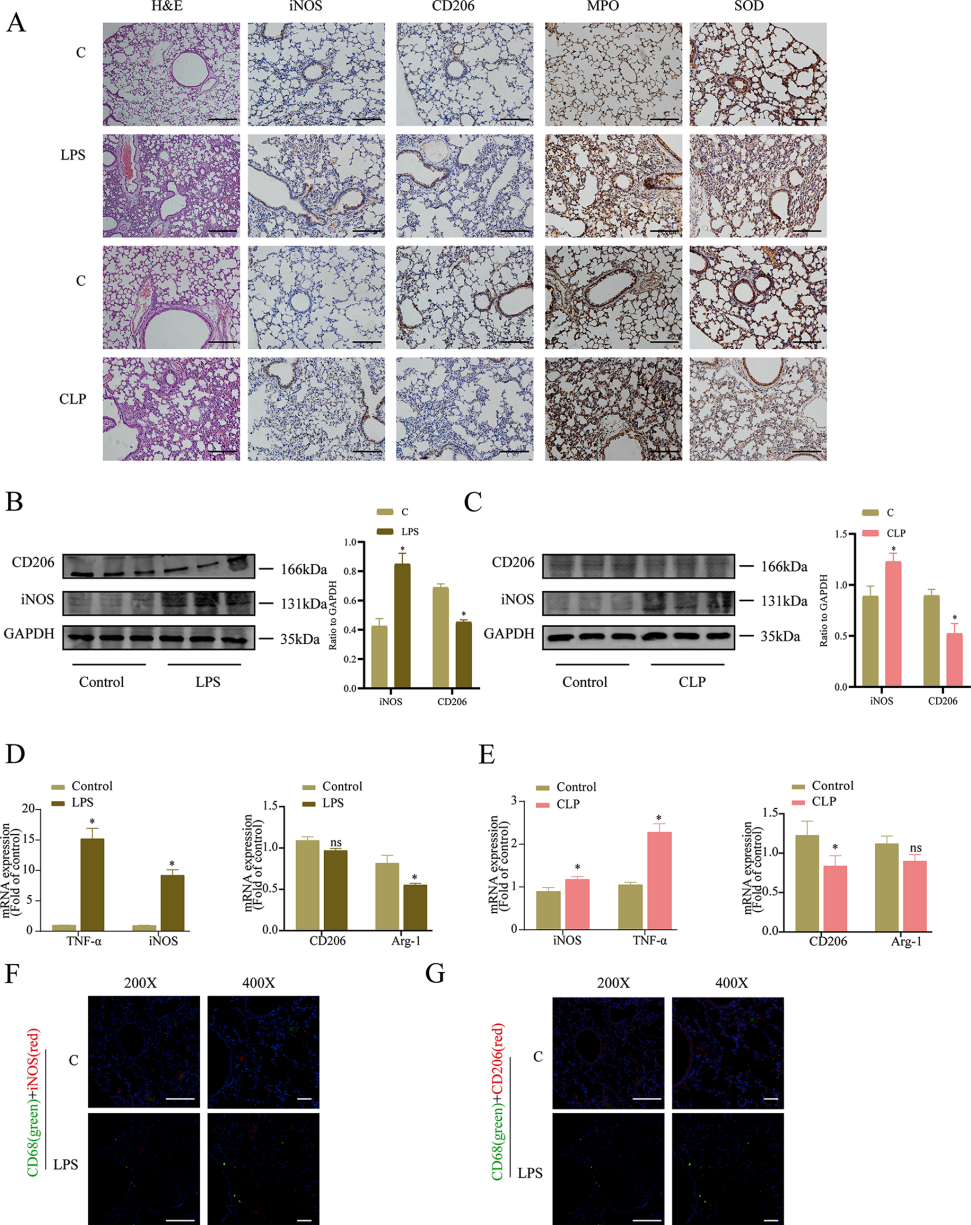
Macrophage polarization occurs in in vivo sepsis models. (**A**) Lung tissue was analyzed by H&E staining, and the expression levels of iNOS, CD206, MPO, and SOD were analyzed by IHC, bar = 50 μm. (**B**) The M1 marker (iNOS) and the M2 marker (CD206) were measured in mice which treated with saline or LPS. (**C**) Relative macrophage polarization related protein expression of M1 marker (iNOS) and M2 marker (CD206) in CLP mice or wild-type mice. (**D**) Quantitative RT-PCR analysis of the M1 markers and the M2 markers in mice treated with LPS. (**E**) Quantitative RT-PCR analysis of the M1 markers and the M2 markers in CLP model. (**F**, **G**) The expression of CD68 in lungs was examined by immunohistochemistry and iNOS, and CD206 level in macrophages (CD68-positive cells) were detected by immunofluorescence. (200×: bar = 50 μm, 400×: bar = 20 μm). ^*^P < 0.05 *vs* the control group. Ns, not significance

Previous studies have demonstrated that macrophages play a crucial role in the immune response during sepsis [4, 5]. To determine the role of macrophage polarization in septic lung injury, we first studied murine endotoxemia and sepsis models. Endotoxemia was induced by intraperitoneal injection and sepsis induced by cecal ligation and puncture in male wild-type mice. In the lungs of endotoxemic and septic mice, there was an increase of macrophages infiltrates, and loss of normal alveolar structure and extensive thickening of the alveolar septa in the lungs of endotoxemic and septic mice (Fig. 1A). IHC staining showed that MPO expression was significantly increased, while SOD1 expression was decreased. Compared to the control group, sepsis model mice exhibited a higher expression level of iNOS (M1 marker) in lungs, but significantly lower expression levels of CD206 (M2 marker) (Fig. 1A). Similarly, the expression of iNOS and TNF-α (M1 markers) was increased in the lung tissues of septic and endotoxemic mice, while the expression of CD206 and Arg-1 (M2 markers) were decreased (Fig. 1B–E). In addition, the infiltrated macrophages were mostly M1-type macrophages, rather than M2 macrophages (Fig. 1F, G). These results strongly suggest that macrophages polarization is involved in the process of sepsis.

### Inflammatory response causes mitochondrial dysfunction in RAW 264.7 cells during macrophages polarization

**Fig. 2:**
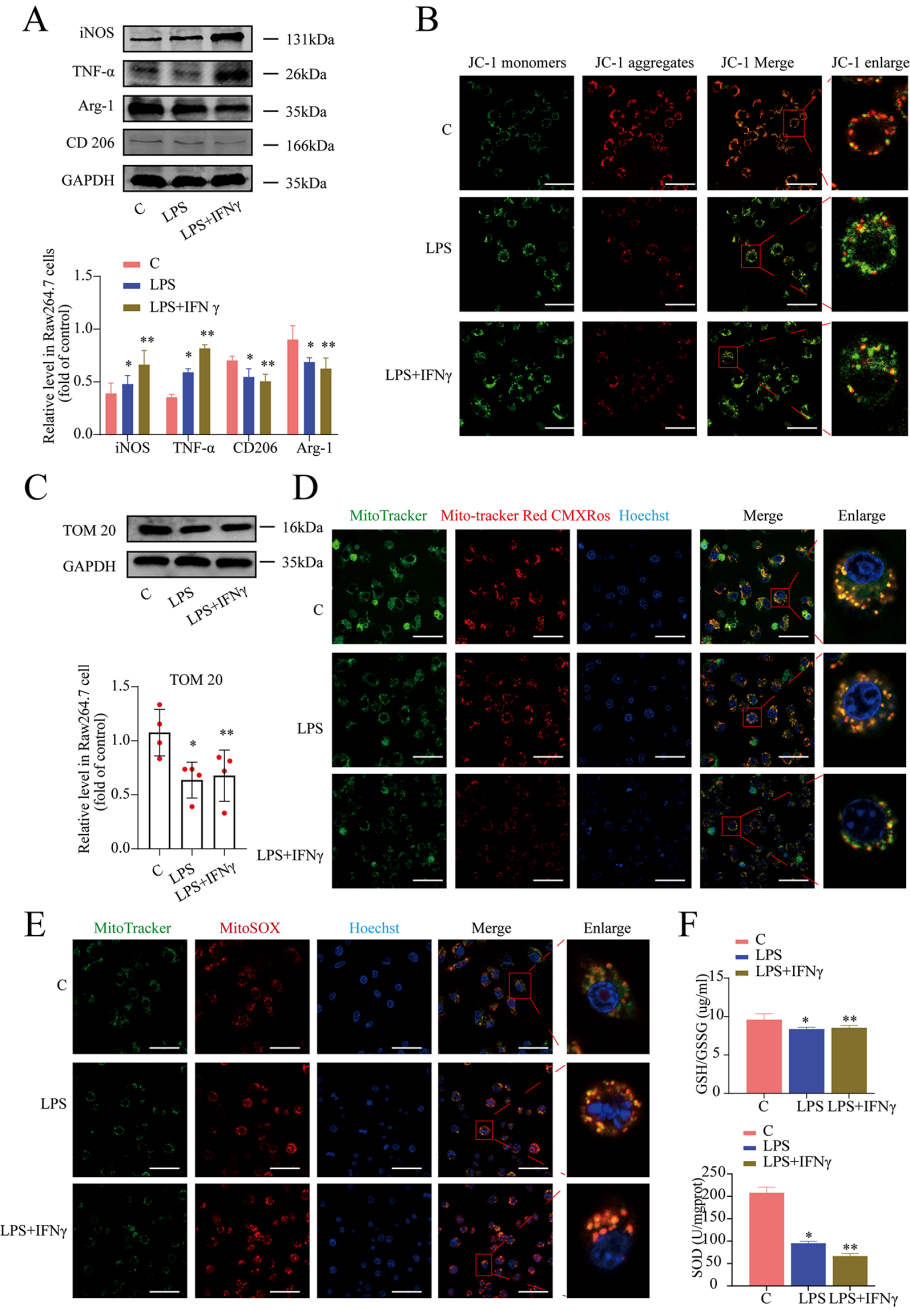
Inflammatory response causes mitochondrial dysfunction in RAW 264.7 cells during macrophages polarization. The RAW 264.7 cells were treated with LPS (100 ng/ml) or LPS + IFN-γ (20 ng/ml) for 24 h. (**A**) The M1 markers (iNOS and TNF-α) and the M2 markers (CD206 and Arg-1) were measured by western blotting. (**B**) The MMP was determined using a JC-1 probe (immunofluorescence straining; scale bar: 10 μm). (**C**) The marker of mitochondria (TOM 20) was detected by western blotting. (**D**) The RAW 264.7 cells were immunostained with 100 nM Mito-Tracker (green) plus 100 nM Mito-Tracker Red CMXRos (red). Quantification of depolarization (damaged) mitochondria. Scale bar: 10 μm. (**E**) Quantification of mitochondrial ROS levels were determined by MitoSOX (red). Scale bar: 10 μm. Meanwhile, (**F**) The level of GSH/GSSG and the level of SOD were measured in RAW 264.7 cells pretreated with LPS or LPS + IFN-γ for 24 h. ^*^P < 0.05, ^**^P < 0.05 *vs* the control group

It has been suggested that mitochondrial oxidative stress and intracellular inflammation are associated with macrophage polarization. To investigate whether mitochondrial dysfunction contributes to macrophage polarization in inflammatory response, we performed Western blotting analysis of RAW 264.7 cells exposed to LPS or LPS plus interferon (IFN)-γ, respectively, to induce macrophages polarization (Fig. 2A). The cells occurred obvious mitochondrial dysfunction, as shown by an increase in JC-1 monomers (green fluorescence) (Fig. 2B). The expression of the mitochondrial marker protein TOM20 in RAW 264.7 macrophage cells was significantly lower than in the control group (Fig. 2C) which meant to inflammatory response could cause the decrease number of mitochondria. Meanwhile, we also did the experiment to observe the mitochondrial transmembrane potential. Changes in mitochondrial potential were determined by Mito Tracker Red CMXRos staining, and the results showed that macrophage polarization reduced the mitochondrial transmembrane potential (Fig. 2D).

Next, an analysis of mitochondrial ROS level in polarized macrophages treated with LPS plus IFN-γ revealed that induction of macrophages polarization led to an increased mitochondrial ROS level (Fig. 2E), a mitochondria-specific superoxide indicator. Consistent with a reduction in mitochondrial oxidative stress, there was a significant reduction of GSH/GSSG and SOD in LPS and LPS plus IFN-γ treated macrophages (Fig. 2F). These results suggested that mitochondrial oxidative stress plays an important role in LPS plus IFN-γ-mediated macrophage polarization.

### Mitochondrial function is required for the macrophage polarization of RAW264.7 cells

**Fig. 3:**
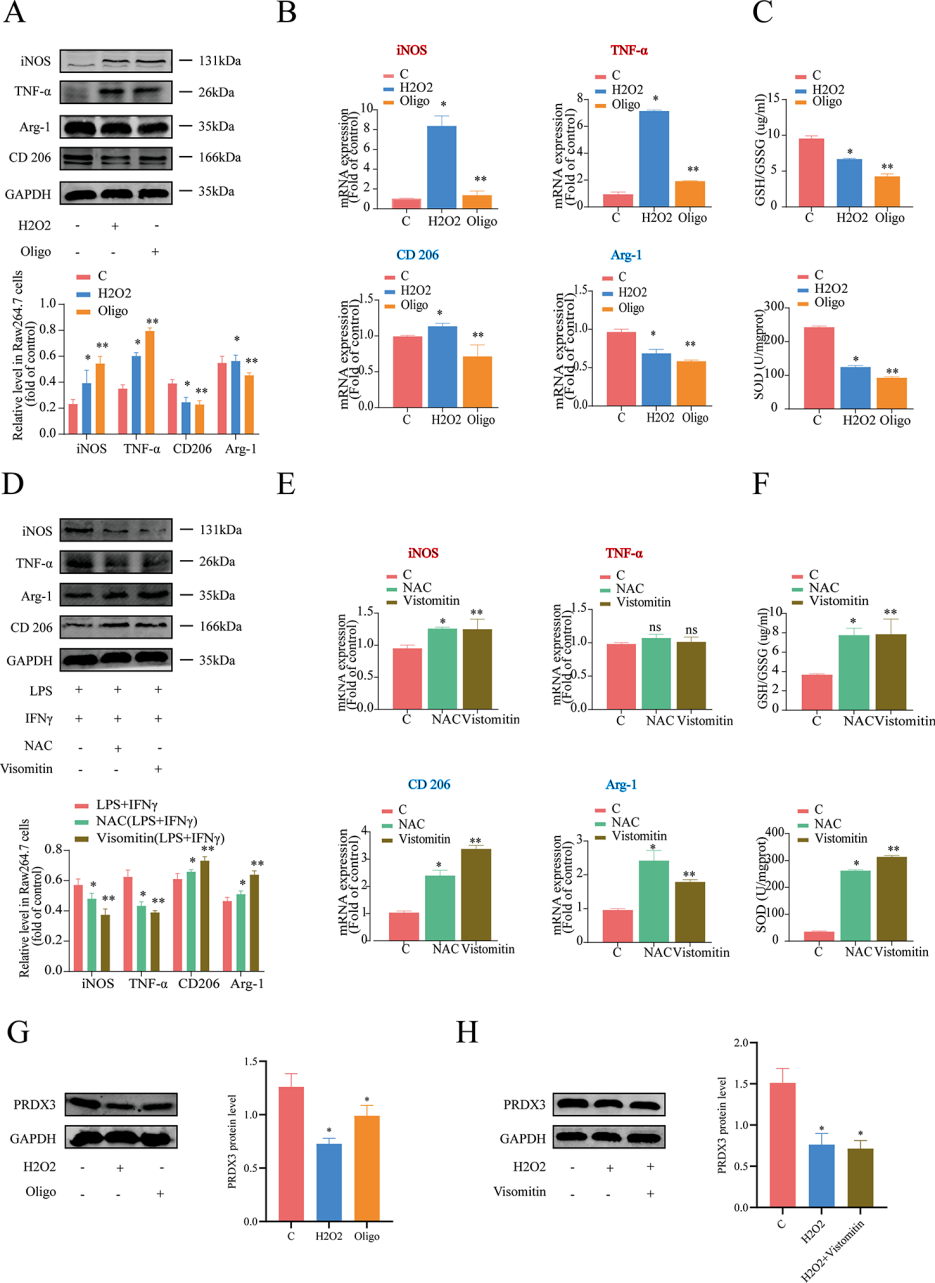
Mitochondrial function is required for the macrophage polarization of RAW264.7 cells. (**A**–**C**) Mitochondrial dysfunction impairs the macrophage polarization of RAW 264.7 cells. (**A**) Relative macrophage polarization related protein expression of M1 markers (iNOS and TNF-α) and M2 markers (CD206 and Arg-1) in cells without (control) or with pretreated with 10 µmol/L H_2_O_2_ or 10 µg/mL Oligo for 24 h (Western blotting). (**B**) Relative macrophage polarization related gene expression of M1 markers (iNOS and TNF-α) and M2 markers (CD206 and Arg-1). (**C**) The level of GSH/GSSG and the level of SOD were measured in RAW 264.7 cells. (**D**–**F**) Reversing mitochondrial dysfunction rescued the macrophage polarization of RAW 264.7 cells. The cells were incubated in medium with both LPS and IFN-γ for 24 h. (**D**) Relative macrophage polarization related protein expression of M1 marker (iNOS and TNF-α) and M2 marker (CD206 and Arg-1) in cells without (control) or with pretreated with 1 mmol/L NAC or 20 nmol/L Visomitin for 2 h (Western blotting). (**E**) Relative macrophage polarization related gene expression of M1 markers (iNOS and TNF-α) and M2 markers (CD206 and Arg-1). (**F**) The level of GSH/GSSG and the level of SOD were measured in RAW 264.7 cells pretreated with NAC or Visomitin after incubated in medium with both LPS and IFN-γ for 24 h. (**G**) Relative PRDX3 expression in RAW 264.7 cells pretreated with 10 µmol/L H_2_O_2_ or 10 µg/mL Oligo for 24 h. (**H**) Relative PRDX3 expression in RAW 264.7 cells pretreated with 20 nmol/L Visomitin for 2 h. ^*^P < 0.05, ^**^P < 0.05 *vs* the control group. Ns: not significance

To elucidate the contribution of mitochondrial function to macrophages polarization, we observed how the changes in the mitochondrial function of cells correlate with macrophage polarization in an environment. RAW 264.7 cell macrophages were pretreated with an ROS generator (10 µmol/L H_2_O_2_) or mitochondrial inhibitor (10 µg/mL oligomycin, Oligo) for 24 h to inhibit mitochondrial function. In addition, other cells were incubated for 2 h with an ROS scavenger (1 mmol/L N-acetylcysteine, NAC) or mitochondrial antioxidant (20 nmol/L visomitin) to enhance the mitochondrial function. The results showed that treatment with either H_2_O_2_ or Oligo significantly decreased the expression of M2-type macrophages-related genes and proteins, while treatment with NAC or visomitin increased the expression of M1-type macrophages-related genes and proteins (Fig. 3A, B). Moreover, a significant reduction of GSH/GSSG and SOD was found in cells treated with either H_2_O_2_ or Oligo (Fig. 3C). In contrast, reversing inflammation-induced mitochondrial dysfunction with NAC or visomitin reversed the effects of H_2_O_2_ and Oligo (Fig. 3D–F). Meanwhile, we found the decrease of PRDX3 in RAW 264.7 cell macrophages was pretreated with H_2_O_2_ or Oligo for 24 h to inhibit mitochondrial function (Fig. 3G). In contrast, cells were pretreated for 2 h with visomitin (Fig. 3H), and the expression of PRDX3 was not increased which meant to M1 macrophage polarization is due to the decreased expression of PRDX3 by ROS. Thus, macrophage polarization of RAW 264.7 cells is closely correlated with mitochondrial function, regardless of whether the cells are incubated in a non-inflammatory or an inflammatory environment.

### Identification and validation of PRDX3 as a key protein associated with macrophage polarization

**Fig. 4:**
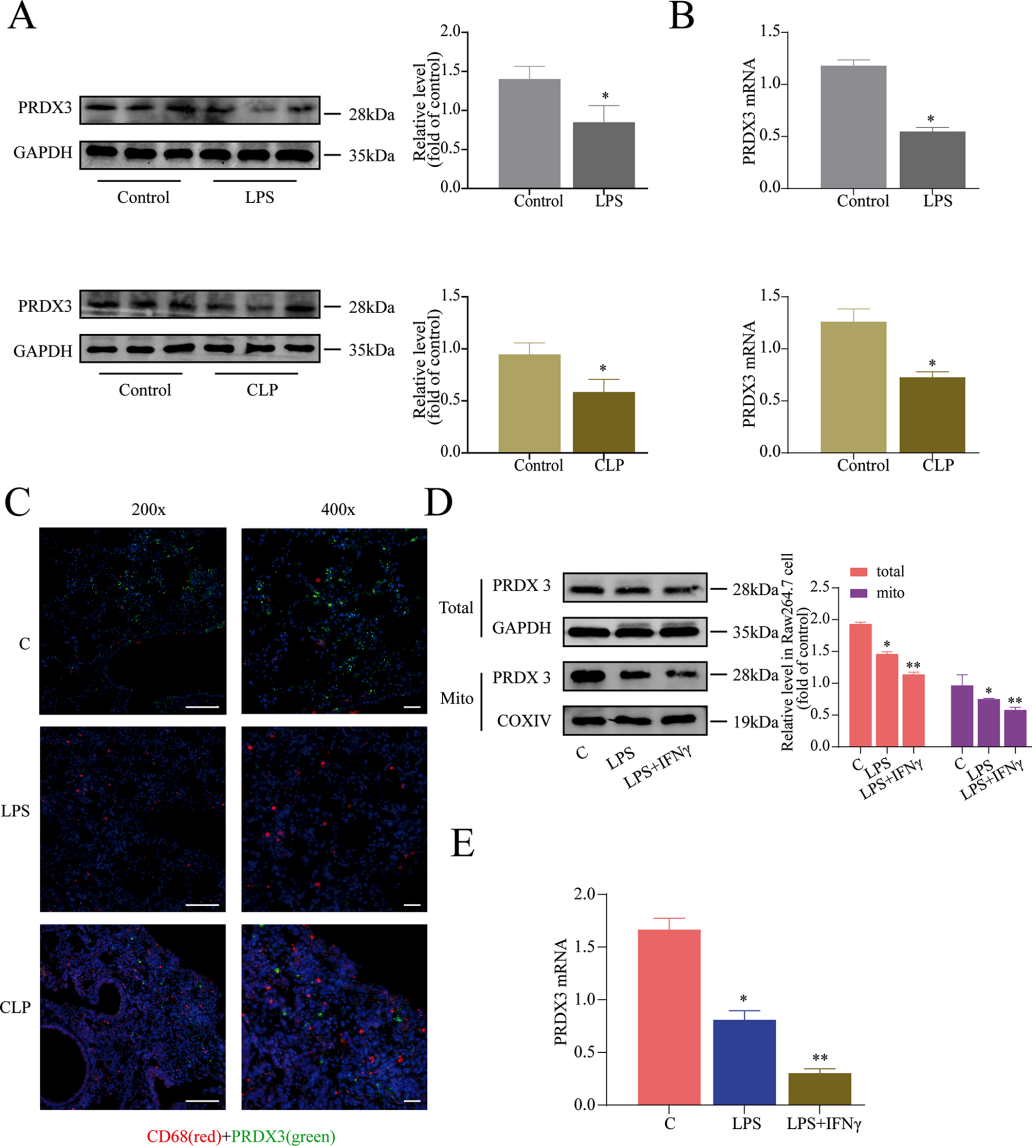
Identification and validation of PRDX3 as a key protein associated with macrophage polarization. (**A**) PRDX3 expression was significantly decreased in lung tissue of LPS-/CLP-treated mice. (**B**) The PRDX3 mRNA expression was analyzed by qPCR. (**C**) The expression of CD68 (red) and PRDX3 (green) in lungs were examined by immunohistochemistry. (200×: bar = 50 μm, 400×: bar = 20 μm) (**D**) Meanwhile, PRDX3 expression was significantly decreased in macrophages. (**E**) The PRDX3 mRNA expression was analyzed by qPCR. ^*^P < 0.05, ^**^P < 0.05 *vs* the control group

Previous studies have demonstrated that PRDX3 is involved in mitochondrial functions, and a lack of PRDX3 results in protection against an inflammatory response, indicating that PRDX3 is involved in the regulation of the inflammatory response [23, 25]. To determine the role of PRDX3 in macrophage polarization during sepsis, we first assessed the expression of PRDX3 in murine endotoxemia and sepsis models. In models of murine sepsis and endotoxemia, the expressions of the PRDX3 gene and protein were decreased in lung tissues (Fig. 4A, B). Thus, PRDX3 is involved in the process of sepsis. We then investigated whether PRDX3 was expressed in macrophages, and the results indicated that the expressions of PRDX3 and CD68 (a macrophage marker) were downregulated in the lung tissue of septic mice (Fig. 4C). Moreover, we also observed that the expression of PRDX3 was decreased in RAW264.7 cells and mitochondria in response to treatment with LPS or LPS plus IFN-γ (Fig. 4D, E). In Addition, we also found the same results in the mouse primary BMDMs after the treatment of LPS/LPS + IFN-γ (Fig. S1). The expression of PRDX3 decreased in the treatment of LPS/LPS + IFN-γ (Fig. S1A). Similarly, the expression of iNOS and TNF-α (M1 markers) was increased in mouse primary BMDMs, while the expression of CD206 and Arg-1 (M2 markers) were decreased (Fig. S1B–D). These results strongly suggest that PRDX3 is involved in macrophage polarization during sepsis.

### Overexpression of PRDX3 reverses inflammation-compromised macrophage polarization and mitochondrial function of RAW 264.7 cells

**Fig. 5:**
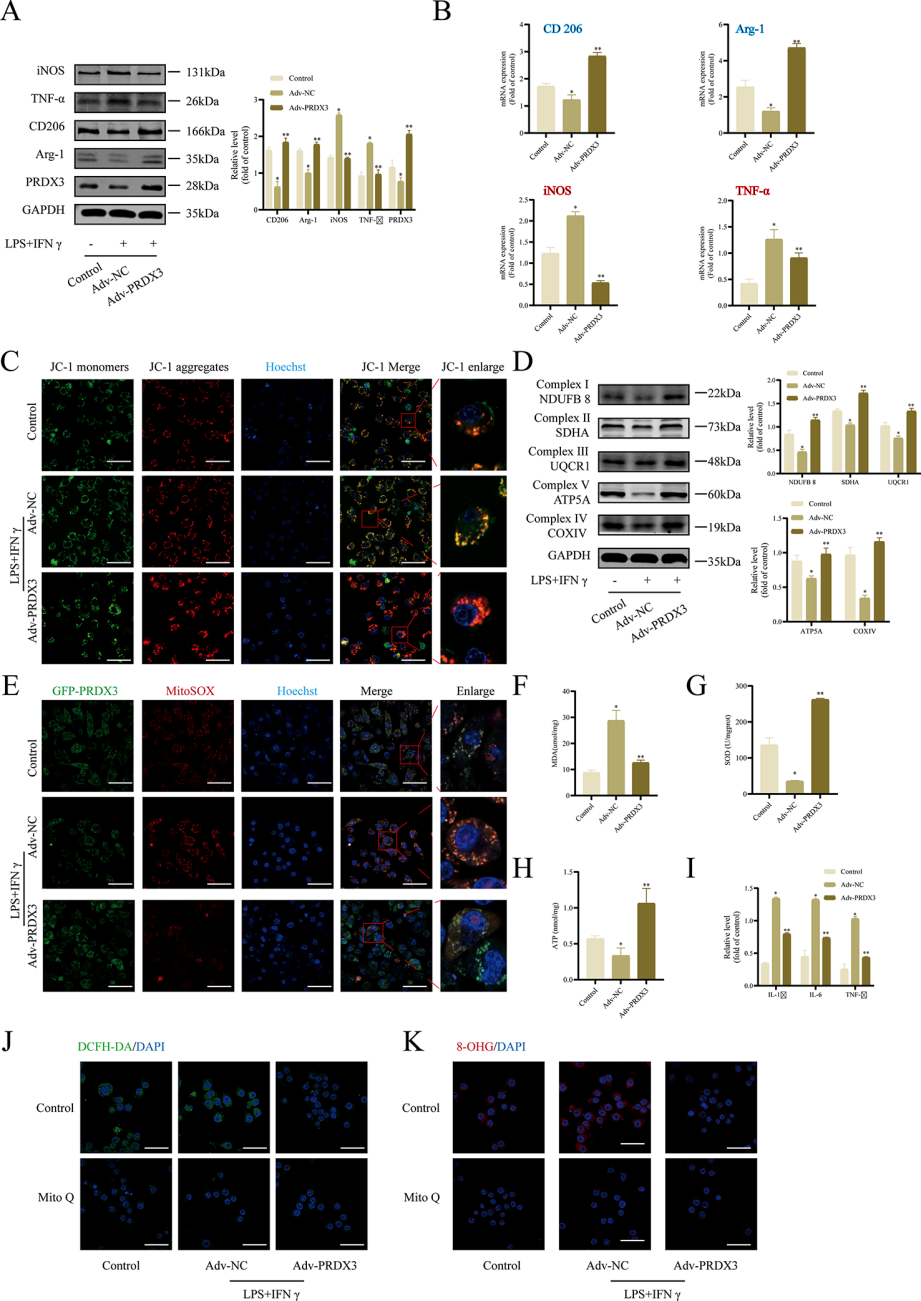
Overexpression of PRDX3 reverses inflammation-compromised macrophage polarization and mitochondrial function of RAW 264.7 cells. The cells were transfected with Adv-NC or Adv-PRDX3 and were incubated in medium with both LPS and IFN-γ for 24 h. The cells were incubated in basal medium in control group. (**A**) Overexpression efficiency of PRDX3 in RAW 264.7 cells validated by western blotting. Meanwhile, relative macrophage polarization related protein expression of M1 markers (iNOS and TNF-α) and M2 markers (CD206 and Arg-1) were detected. (**B**) Relative macrophage polarization-related gene expression level of CD206, Arg-1, iNOS, and TNF-α determined by qRT-PCR. (**C**) The MMP was determined using a JC-1 probe (immunofluorescence straining; scale bar: 10 μm). (**D**) Relative mitochondrial complex-related protein expression of NDUFB8 (subunit of complex I), SDHA (subunit of complex II), UQCR1 (subunit of complex III), COXIV (subunit of complex IV) and ATP5A (subunit of complex V) determined by western blotting. (**E**) Quantification of mitochondrial ROS levels were determined by MitoSOX (red) in various groups. Scale bar: 10 μm. (**F**) The level of MDA and the level of SOD (**G**) were measured in RAW 264.7 cells transfected with Adv-NC or Adv-PRDX3 and then incubated in medium with both LPS and IFN-γ for 24 h. (**H**) The level of ATP was measured. (**I**) The level of inflammatory factors (IL-6/IL-1β/TNF-α) was measured. (**J**) ROS staining in macrophages after PRDX3 and MitoQ interference. Scale bar: 10 μm. (**K**) 8-OHG staining in macrophages after PRDX3 and MitoQ interference. Scale bar: 10 μm. ^*^P < 0.05, *vs* the Control group, ^**^P < 0.05, *vs* the Adv-NC group

To further understand the effect of PRDX3 on macrophage polarization during sepsis, we overexpressed PRDX3 in RAW 264.7 cells with lentivirus carrying PRDX3 cDNA (Adv-PRDX3) (Fig. 5A), and incubated the stable overexpression cells in medium with both LPS and IFN-γ. Compared with Adv-NC transfection, Adv-PRDX3 transfection led to significantly higher CD206 and Arg-1 protein expression levels in RAW 264.7 cells (Fig. 5A), as well as significantly higher CD206 and Arg-1 gene expression levels (Fig. 5B). In addition, expressions of M1-type macrophage-related genes and proteins (iNOS and TNF-α) were decreased (Fig. 5A, B). Moreover, PRDX3 overexpression alleviated the mitochondrial dysfunction, as shown by a decrease in JC-1 monomers (green fluorescence) (Fig. 5C), and increased the expression of mitochondrial respiratory chain complex I/II/III/IV/V (Fig. 5D). Similarly, mitochondrial ROS levels decreased significantly following PRDX3 overexpression (Fig. 5E). Consistently, PRDX3 overexpression decreased the MDA level (Fig. 5F) and inflammatory cytokine (Fig. 5I), and increased SOD (Fig. 5G) and ATP (Fig. 5H) levels in RAW 264.7 cells.

Furthermore, we performed rescue experiments with RAW 264.7 cells using the antioxidant mitochondria-targeted ROS scavenger mitoquinone (MitoQ) to examine whether PRDX3 could alleviate the mitochondria-targeted ROS. MitoQ abrogated the increased levels of ROS and oxidative DNA damage (Fig. 5J, K). Together, these data suggest that PRDX3 overexpression can reverse macrophage polarization and mitochondrial dysfunction of RAW 264.7 cells.

### PRDX3 overexpression induces metabolic reprogramming in RAW 264.7 cells

**Fig. 6:**
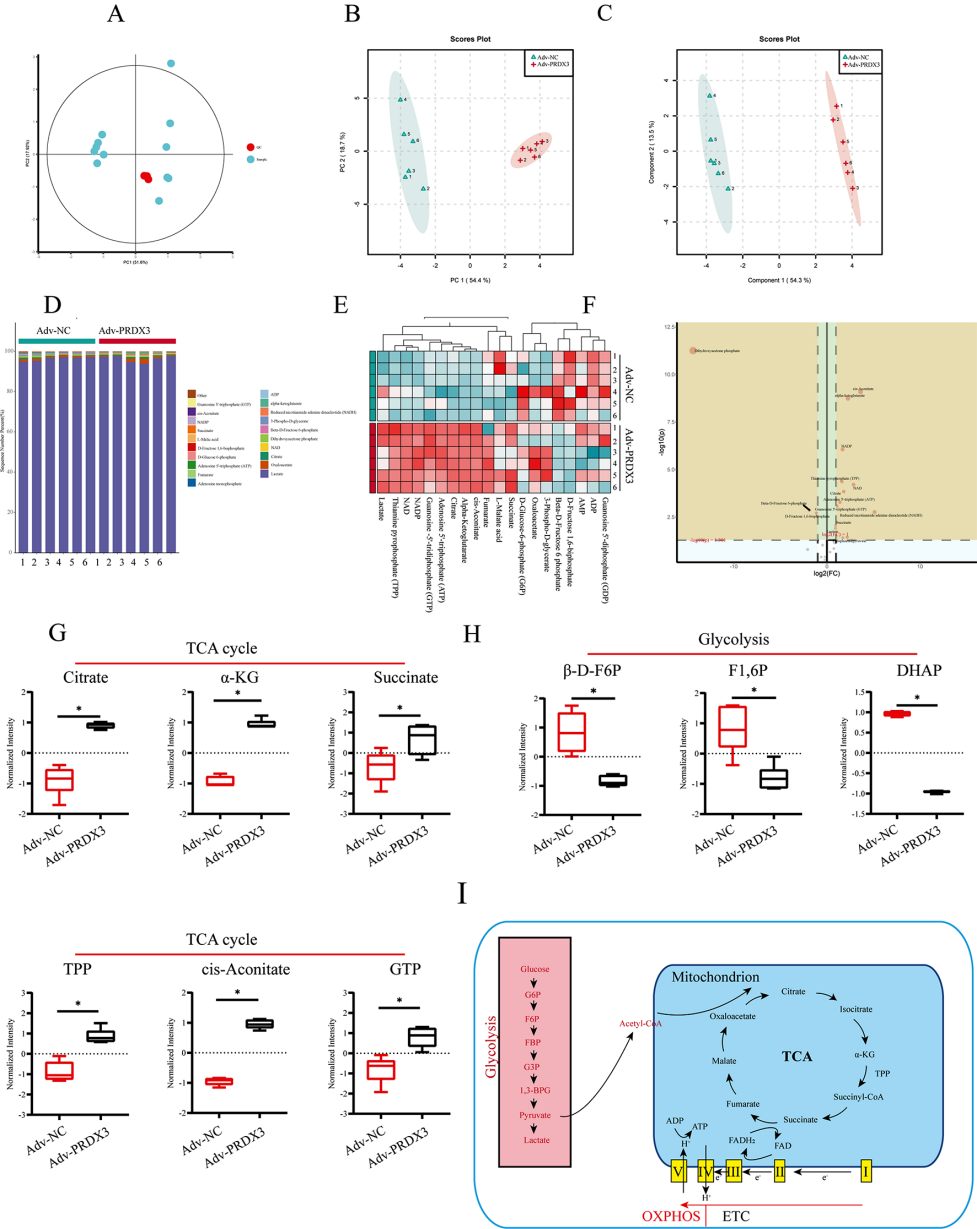
PRDX3 overexpression induces metabolic reprogramming in RAW 264.7 cells. (**A**) QC, (**B**) PCA and (**C**) PLS-DA comparing subjects from Adv-NC and Adv-PRDX3 groups. (**D**) The structural composition of the metabolites in Adv-NC and Adv-PRDX3 group. (**E**) The abundance of Top 20 metabolites in Adv-NC and Adv-PRDX3 group. (**F**) Volcano of 25 metabolites in Adv-NC and Adv-PRDX3 group. (**G**, **H**) The levels of Citrate, α-KG, Succinate, TPP, cis-Aconitate, GTP, β-D-F6P, F1,6P and DHAP detected in RAW 264.7 cells after PRDX3 overexpression. (**I**) Schematic diagram of Glycolysis and the TCA cycle. ^*^P < 0.05, *vs* the Adv-NC group

We next explored if PRDX3 can drive metabolic reprogramming to reverse macrophage polarization. Targeted metabolomics was performed to explore the effects of PRDX3 on the improvement of metabolic reprogramming. First, the quality control (QC) sample was found to be closely clustered together (Fig. 6A), indicating good repeatability of this experiment. This indicates that the experimental data are stable and reliable between the 2 groups of samples. The principal components analysis (PCA) score plot shows the distribution between the 2 groups in 2D space (Fig. 6B). Samples from the same group are almost clustered together, and within the 95% confidence interval (CI), indicating the variables observed in the samples are biologically relevant.

Partial least squares discriminant analysis (PLS-DA) was performed on cell samples. Significant clustering was observed among the Adv-NC and Adv-PRDX3 groups, indicating that inter-group differences were more significant than individual differences (Fig. 6C). Based on the above results, the structural composition of the metabolites was analyzed in the Adv-NC and Adv-PRDX3 groups (Fig. 6D). To further explore the differences in metabolites, the top 20 significantly changed metabolites were analyzed (Fig. 6E). Potential metabolites that contributed significantly to clustering and differentiation were selected based on their variable importance projection (VIP) values and *P*-values (Fig. 6F). The metabolic assay results showed that PRDX3 overexpression increased the levels of several tricarboxylic acid cycle (TCA) cycle metabolites in RAW 264.7 cells treated with LPS and IFN-γ (Fig. 6G), while overexpression decreased the levels of several glycolysis-related metabolites (Fig. 6G), indicating that PRDX3 can drive metabolic reprogramming in macrophage polarization. Meanwhile, we also found the same results in the cells after repressing glycolysis. 3PO, inhibitor repressing PFKFB3, could counteract the pro-inflammatory effect of LPS (Fig. S2). The expression of iNOS and TNF-α (M1 markers) was decreased in LPS + 3PO group (Fig. S2A, B), while the expression of CD206 and Arg-1 (M2 markers) were increased (Fig. S2A, C). Taken together, the results indicate that PRDX3 overexpression causes M1-type macrophages to differentiate into M2 macrophages, which has the potential to reverse the macrophage polarization.

**Fig. 7:**
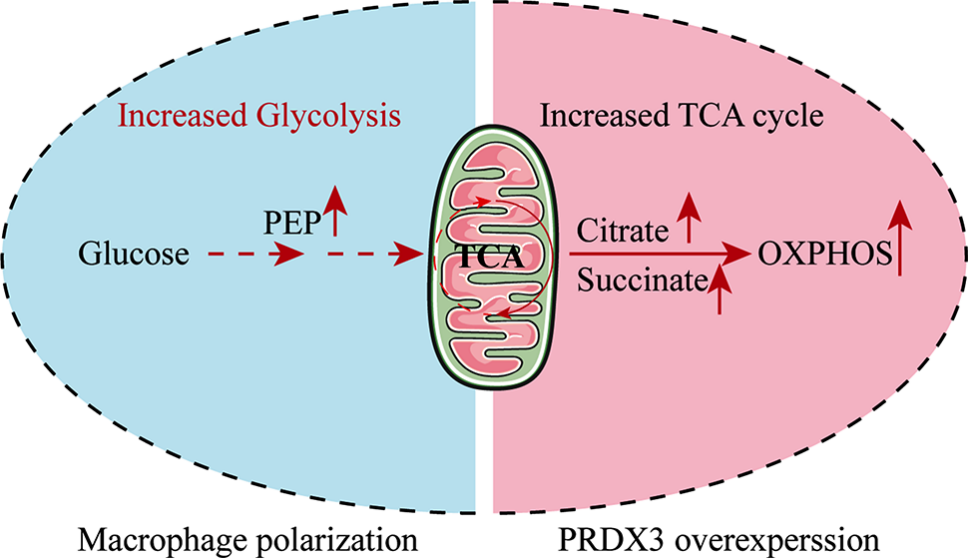
Schematic diagram of the role of PRDX3 in protecting macrophages polarization in sepsis-associated ALI

In this study, we investigated whether PRDX3 reversed macrophage polarization by inducing metabolic reprogramming, and found that PRDX3 overexpression increased the levels TCA cycle metabolites and decreased the levels of glycolysis-related metabolites after injury (Fig. 7).

## Discussion

In this study we investigated whether PRDX3 deficiency could accelerate macrophage polarization in septic lung injury. The main findings of this study are: (1) Macrophage polarization occurs in septic lung injury as a result of mitochondrial dysfunction. (2) PRDX3 deficiency activates macrophages and accelerates inflammation in RAW 264.7 cell macrophage. (3) PRDX3 overexpression promotes M1-type macrophages to differentiate into M2 macrophages, and enhanced mitochondrial function recovery after injury by reducing the level of glycolysis and increasing the TCA cycle level. These findings suggest that PRDX3 may be a new therapeutic target with respect to macrophage polarization in septic lung injury.

Inflammation is a leading cause of sepsis-induced lung injury, and macrophages play significant roles in the immunopathogenesis of sepsis [26, 27]. Previous studies have demonstrated that excessive macrophages can induce tissue damage during inflammation [28, 29], and macrophage polarization can be induced during sepsis [30, 31]. Macrophages, as the first line of defense, trigger an immune response in the lungs and macrophage polarization gives rise to a wide range of functional phenotypes [32]. In the inflammatory response, macrophages are classified into classically activated macrophages (M1) under the stimulation of LPS or IFN-γ. M1 macrophages secrete inflammatory cytokines, including IL-1β, IL-6, TNF-α, and CXCL-10 to enhance antimicrobial activity and the recruitment of the immune cells in the lung [11]. In comparison, M2 macrophages have anti-inflammatory functions for the resolution of inflammation and healing of tissues [33]. However, how macrophage polarization works in sepsis or septic lung injury is not completely known.

In this study, we explored the mechanism of macrophage polarization in septic lung injury and demonstrated that increased M1 macrophages and decreased M2 macrophages are observed in progression of septic lung injury. Previous studies have shown that mitochondrial functions play a crucial role in macrophage polarization [34–36]. The results of our study also illustrated the role of mitochondrial dysfunction in the imbalance between M1 and M2 macrophage polarization. The experimental results indicated that an inflammatory environment caused mitochondrial dysfunction in macrophages, which is consistent with previous findings. On the other hand, we found that reversing inflammation-induced mitochondrial dysfunction reversed the effect of inflammation on macrophage polarization. These data indicate that the macrophage mitochondrial dysfunction is a primary cause of macrophage polarization, and thus provides new understanding for the imbalance between M1 and M2 macrophage polarization in septic lung injury.

PRDX3 is located in the mitochondria, and plays a crucial role in the reduction and clearance of peroxynitrite [25]. Research has shown that a deficiency of PRDX3 leads to an increase in mitochondrial superoxide levels, and the induction of inflammation [21, 22]. Conversely, overexpression of PRDX3 effectively scavenges excess H_2_O_2_ and preserves mitochondrial function against mitochondrial H_2_O_2_ [37]. In this study, we identified there is a deficiency in PRDX3 in septic lung injury, which is accompanied by an imbalance between M1 and M2 macrophage polarization.

Additionally, our findings demonstrated that overexpression of PRDX3 in RAW 264.7 cell macrophages effectively reduced mitochondrial H_2_O_2_ and superoxide levels. Importantly, our results indicate an association between PRDX3 and the regulation of mitochondrial respiratory chain complex I/II/III/IV/V, and we demonstrated that PRDX3 overexpression has the potential to convert M1 macrophages to M2 macrophages. Notably, we investigated the mechanism by which PRDX3 regulates mitochondrial dysfunction and macrophage polarization. Mitochondrial function has been reported in the macrophage polarization and inflammation [17]. Mitochondrial-related cellular metabolism also plays an important role in macrophage polarization [38, 39]. The activation of M1 macrophages by various inflammatory stimuli induces metabolic alternations, especially the upregulation of aerobic glycolysis, accompanied by an impaired respiratory chain and the production of ROS. Inhibition of glycolysis has been shown to alleviate inflammation, leading to alleviation of ALI in mice [40]. Furthermore, a truncated TCA cycle is another metabolic feature of M1 macrophages, which also promotes inflammation [41–43]. However, the specific target or mechanism of action is not yet clear. In this study, we found the PRDX3 plays an important role in inflammation and macrophage polarization, and PRDX3 is markedly reduced in septic lung injury. In addition, our findings revealed that PRDX3 overexpression leads to an increase in TCA cycle metabolites (citrate, α-KG, succinate, TPP, cis-aconitate, and GTP) and a decrease in glycolysis-related metabolites (β-D-F6P, F 1,6P, and DHAP), as shown by targeted metabolomics analysis. These findings indicate the crucial role of PRDX3 in regulating mitochondrial function and macrophage polarization.

In conclusion, our data demonstrate for the first time a pivotal role of PRDX3 in regulating mitochondrial function and macrophage polarization in septic lung injury. Overexpression of PRDX3 can reverse inflammation-compromised mitochondrial function and macrophage polarization. These findings suggest that PRDX3 may be a novel target for the treatment of septic lung injury.

### Electronic supplementary material

Below is the link to the electronic supplementary material.

**Supplementary Fig. 1** Inflammatory response causes macrophages polarization and decreases the expression of PRDX3 in BMDMs. The mouse primary BMDMs cells were treated with LPS (100 ng/ml) or LPS + IFN-γ (20 ng/ml) for 24 h. (**A**) The expression of PRDX3 was measured by western blotting. (**B**) The M1 markers (iNOS and TNF-α) and the M2 markers (CD206 and Arg-1) were measured by western blotting. (**C**) Relative macrophage polarization related gene expression of M1 markers (iNOS and TNF-α). (**D**) Relative macrophage polarization related gene expression of M2 markers (CD206 and Arg-1). ^*^P < 0.05, ^**^P < 0.05 *vs* the control group. **Supplementary Fig. 2** Repressing glycolysis could regulate M1/M2 differentiation. The Raw264.7 were treated with LPS (100 ng/ml) or LPS + 3PO (10 µmol/L) for 24 h. (**A**) The expression of M1-/M2- type macrophages was measured by western blotting. (**B**) Relative macrophage polarization related gene expression of M1 markers (iNOS and TNF-α). (**C**) Relative macrophage polarization related gene expression of M2 markers (CD206 and Arg-1). ^*^P < 0.05, *vs* the control group, ^**^P < 0.05 *vs* the LPS group

## Data Availability

The data underlying this article will be shared on reasonable request to the corresponding author.

## Abbreviations

α-KG: α-ketoglutarate
TPP: thiamine pyrophosphate
GTP: Guanosine − 5’-tridiphosphate
β-D-F6P: beta-D-fructose-6-phosphate
F 1,6P: fructose-1,6-phosphate
DHAP: dihydroxyacetone phosphate
ALI: Acute lung injury
ROS: reactive oxygen species
LPS: lipopolysaccharide
TCA: tricarboxylic acid cycle
PRDXs: Peroxiredoxins
