# Supplementary material for: Peroxiredoxin 3 has a crucial role in the macrophage polarization by regulating mitochondrial homeostasis
Source: Respir Res. 2024 Mar 2;25:110. doi: 10.1186/s12931-024-02739-9 (PMC10909251; doi:10.1186/s12931-024-02739-9)
Supplement: Supplementary file 1 — Supplementary Fig. 1 Inflammatory response causes macrophages polarization and decreases the expression of PRDX3 in BMDMs. The mouse primary BMDMs cells were treated with LPS (100 ng/ml) or LPS + IFN-γ (20 ng/ml) for 24 h. (A) The expression of PRDX3 was measured by western blotting. (B) The M1 markers (iNOS and TNF-α) and the M2 markers (CD206 and Arg-1) were measured by western blotting. (C) Relative macrophage polarization related gene expression of M1 markers (iNOS and TNF-α). (D) Relative macrophage polarization related gene expression of M2 markers (CD206 and Arg-1). *P < 0.05, **P < 0.05 vs the control group. Supplementary Fig. 2 Repressing glycolysis could regulate M1/M2 differentiation. The Raw264.7 were treated with LPS (100 ng/ml) or LPS + 3PO (10 µmol/L) for 24 h. (A) The expression of M1-/M2- type macrophages was measured by western blotting. (B) Relative macrophage polarization related gene expression of M1 markers (iNOS and TNF-α). (C) Relative macrophage polarization related gene expression of M2 markers (CD206 and Arg-1). *P < 0.05, vs the control group, **P < 0.05 vs the LPS group [file 12931_2024_2739_MOESM1_ESM.docx]

**Peroxiredoxin 3 has a crucial role in the Macrophage Polarization by regulating mitochondrial homeostasis**

Wenhui Huang^2, #^, Lianfang Wang^3, #^, Zhipeng Huang^4^, Zhichao Sun^5^, Bojun Zheng^1, *^

^1^Department of Critical Care Medicine, The Second Affiliated Hospital of Guangzhou University of Chinese Medicine, Guangzhou, China.

^2^Department of Respiratory and Critical Care Medicine; Guangdong Provincial Key Laboratory of Major Obstetric Diseases; Guangdong Provincial Clinical Research Center for Obstetrics and Gynecology; The Third Affiliated Hospital of Guangzhou Medical University, Guangzhou, China.

^3^Department of Respiratory and Critical Care Medicine, Guangxi Hospital Division of The First Affiliated Hospital, Sun Yat-sen university, Guangxi, China.

^4^Dongguan Hospital of Integrated Chinese and Western Medicine, Dongguan, China.

^5^The Second Affiliated Hospital of Guangzhou University of Chinese Medicine, Guangzhou, China.

^#^These authors contributed equally to this paper.

^*^Correspondence: Bojun Zheng, Department of Critical Care Medicine, The Second Affiliated Hospital of Guangzhou University of Chinese Medicine, Guangzhou, China. Email: [zhengbojun@gzucm.edu.cn](mailto:zhengbojun@gzucm.edu.cn).

**Supplementary Figures and legends**


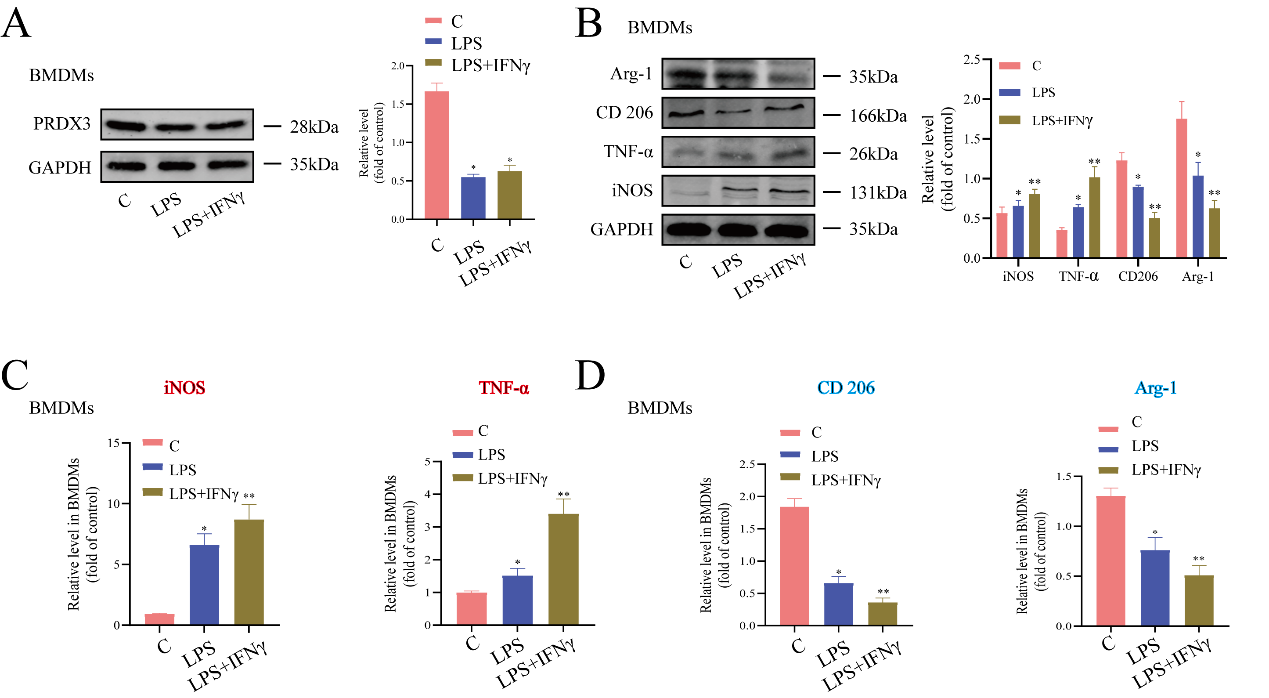


**Supplemental Figure 1**

**Supplementary Figure 1. Inflammatory response causes macrophages polarization and decreases the expression of PRDX3 in BMDMs.**

The mouse primary BMDMs cells were treated with LPS (100 ng/ml) or LPS+ IFN-γ (20 ng/ml) for 24 hours. **(A)** The expression of PRDX3 was measured by western blotting. **(B)** The M1 markers (iNOS and TNF-α) and the M2 markers (CD206 and Arg-1) were measured by western blotting. **(C)** Relative macrophage polarization related gene expression of M1 markers (iNOS and TNF-α). **(D)** Relative macrophage polarization related gene expression of M2 markers (CD206 and Arg-1). ^*^*P*< 0.05, ^**^*P* < 0.05 *vs* the control group.


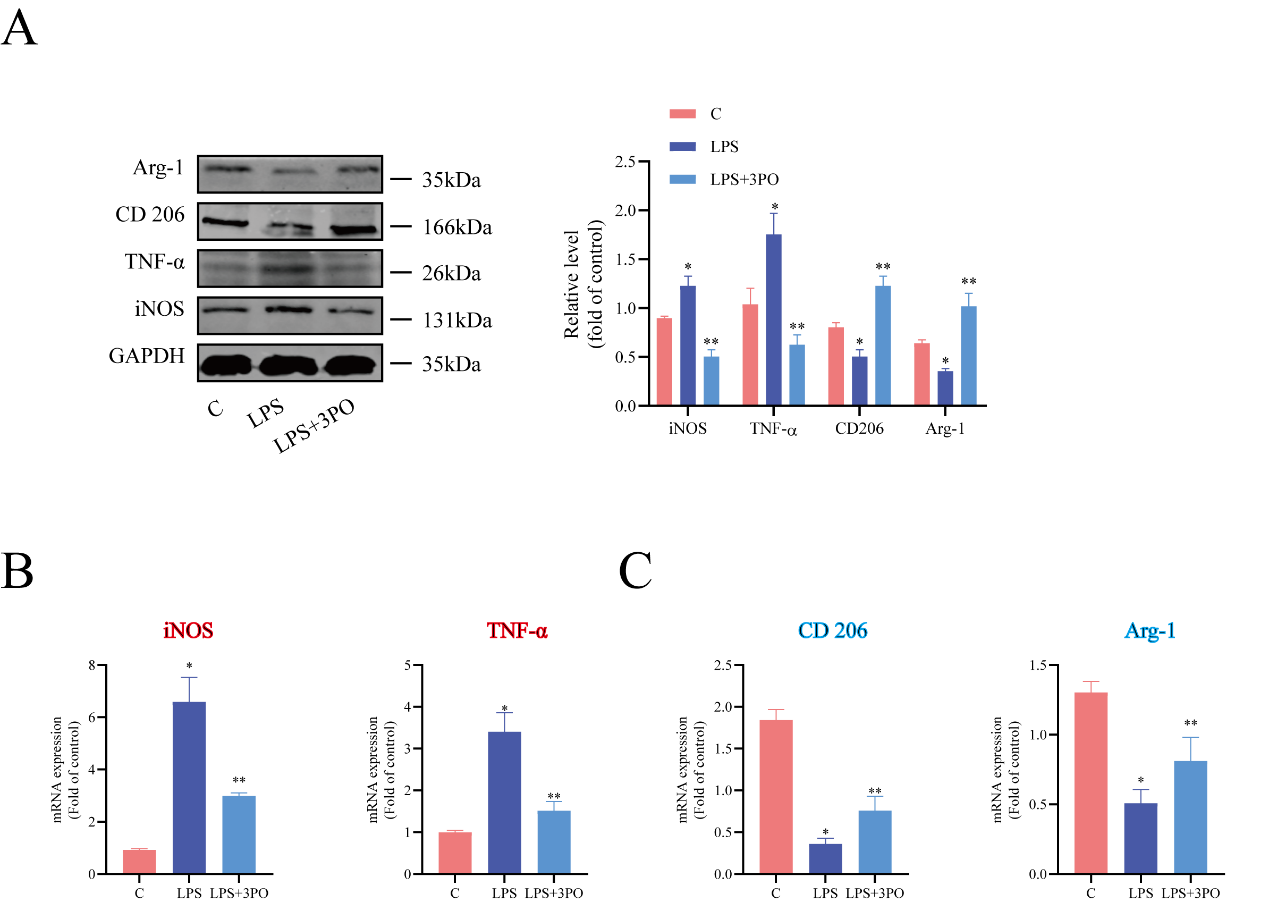


**Supplemental Figure 2**

**Supplementary Figure 2. Repressing glycolysis could regulate M1/M2 differentiation.**

The Raw264.7 were treated with LPS (100 ng/ml) or LPS+ 3PO (10 µmol/L) for 24 hours. **(A)** The expression of M1-/M2- type macrophages was measured by western blotting. **(B)** Relative macrophage polarization related gene expression of M1 markers (iNOS and TNF-α). **(C)** Relative macrophage polarization related gene expression of M2 markers (CD206 and Arg-1). ^*^*P*< 0.05, *vs* the control group, ^**^*P* < 0.05 *vs* the LPS group.
